# Exploring Aubrac Cattle as a Benchmark for Sustainable and Nutritious Beef Production

**DOI:** 10.3390/ani15202966

**Published:** 2025-10-13

**Authors:** Bianca Maria Mădescu, Mădălina Matei, Mădălina Alexandra Davidescu, Ioana Bolohan (Acornicesei), Roxana Lazăr, Marius Mihai Ciobanu, Daniel Simeanu, Paul Corneliu Boișteanu

**Affiliations:** “Ion Ionescu de la Brad” Iasi University of Life Sciences, 3 Mihail Sadoveanu Alley, 700489 Iasi, Romania

**Keywords:** Aubrac cattle, amino acids, fatty acids, meat quality, protein value

## Abstract

**Simple Summary:**

Beef quality is an important factor for both consumers and farmers, as it combines taste, tenderness, and nutritional value. The Aubrac cattle breed, originally from France and now increasingly raised in Eastern Europe, is known for producing flavorful meat, but little is known about its nutritional characteristics. In this study, we analyzed the meat of Aubrac cattle to better understand its protein and fat composition. We found that the meat provides all the essential amino acids required for healthy growth and development, in amounts that go beyond international recommendations for children, youth, and adults. In addition, the meat contains a favorable balance of fats, with a high level of oleic acid, which is considered beneficial for heart health. These results show that Aubrac beef is not only tender and tasty but also a valuable and healthy source of protein. This information can help consumers make informed dietary choices, encourage farmers to adopt sustainable production systems, and support the promotion of high-quality beef that contributes to human health and well-being.

**Abstract:**

The Aubrac cattle breed, native to France and increasingly adopted in Eastern Europe, is known for producing tender, flavorful, and well-marbled beef. Despite its growing popularity, limited data exist on its nutritional profile and implications for human health. This study evaluates carcass traits and protein quality in the *Longissimus dorsi* muscle of male and female Aubrac cattle raised under semi-intensive systems. Special attention was given to essential and semi-essential amino acids, which exceeded FAO/WHO reference requirements by 60.25%, 97.43%, and 221.49% for children, youth individuals, and adults, respectively, confirming superior protein quality. The high amino acid concentration (up to 30.59 g/100 g protein) and biological value confirm superior protein quality. Furthermore, the fatty acid profile reveals a favorable balance between saturated and unsaturated fats, with oleic acid predominance and a moderate atherogenic index, suggesting cardiovascular benefits. These findings support classifying Aubrac beef as a functional protein source with potential health advantages, reinforcing its role in sustainable cattle production, dietary planning, and consumer education. This study provides insights into factors influencing beef quality, connecting cattle breed and production system with nutritional value and health benefits.

## 1. Introduction

In recent years, there has been growing interest in understanding the nutritional composition of beef and its impact on human health [1,2]. As consumers become more conscious of their dietary choices, exploring beef varieties with desirable nutritional profiles has become increasingly important [2]. One emerging trend in Romanian husbandry is the breeding of Aubrac cattle, originally from the Aubrac region in France, valued for superior meat quality and adaptability to diverse farming conditions. Compared with established beef breeds such as Charolais and Angus, Aubrac cattle demonstrate remarkable adaptability, thriving in diverse environments in Romania, including hilly, mountainous, and plain areas [3]. The breeding of Aubrac cattle exemplifies diversification in livestock systems, offering economic benefits through cost-efficient production and ecological advantages by promoting pasture biodiversity and soil health. This trend benefits both farmers and consumers, providing high-quality meat with distinctive characteristics [4]. Aubrac beef is prized for its fine texture, juiciness, excellent marbling, and high content of healthy fats, making its nutritional composition of considerable interest to both producers and consumers [3,4].

Beef consumption contributes to a balanced and nutritious diet, being a rich source of high-quality proteins, fatty acids, essential amino acids, vitamins, and minerals [5,6]. Proteins and lipids, as major nutritional components of animal feed, play a decisive role in livestock growth, tissue development, and ultimately meat quality [7,8,9,10]. Proteins, particularly amino acids, are crucial for muscle development and contribute to both yield and nutritional value. Lipids influence fat deposition, including intramuscular fat or marbling, which improves tenderness, juiciness, and flavor [9,11]. Thus, a balanced diet that provides adequate amounts of proteins and lipids ensures optimal growth and desirable meat characteristics [8,9,10,11]. The fatty acid composition, especially the ratio of omega-3 to omega-6, affects the sensory qualities and nutritional profile of beef [6,12]. Beef contains saturated (SFAs), monounsaturated (MUFAs), and polyunsaturated fatty acids (PUFAs) [13]. Unsaturated fats are beneficial for health, whereas high levels of SFA, particularly C12:0 (lauric acid), C14:0 (myristic acid), and C16:0 (palmitic acid), are associated with cardiovascular disease. Dietary lipids, typically 1–4% of cattle feed and largely composed of PUFA, are biohydrogenated by rumen microorganisms into less hazardous SFA, notably 18:0 (stearic acid) [13,14,15]. The abundant fatty acids in beef are C18:1 cis (oleic acid), C18:0 (stearic acid), and C16:0 (palmitic acid) [5,8]. Feeding strategies influence fatty acid profiles: pasture-based diets favor unsaturated fats, whereas concentrate-rich diets increase SFA content [16,17,18,19]. Beef is also a rich source of amino acids, including phenylalanine, isoleucine, histidine, leucine, lysine, methionine, threonine, tryptophan, and valine [1,20], which support protein synthesis, tissue repair, enzyme production, and immune function. Overall, beef contains approximately 20–25% essential amino acids, making it a rich source of high-quality protein [17,21]. The amino acid profile can vary with diet, and nutrient-rich feeding enhances meat nutritional value.

The diet of animals is a key factor in ensuring that beef delivers its full potential, highlighting the importance of sustainable and well-managed feeding practices [19,22]. Meat quality is influenced by both genetic factors (breed, age, sex, muscle type) and external factors such as diet and farm management [23]. A semi-intensive system offers benefits for animal welfare, meat quality, environmental sustainability, and food safety compared to intensive systems [24,25,26]. Moreover, natural and diverse diets, such as grazing on fresh pasture and herbs, improve fat profile by increasing omega-3 fatty acids and essential vitamins, resulting in healthier and higher-quality meat for consumers [27,28]. We hypothesized that gender influences the fatty acid and amino acid composition of the *Longissimus dorsi* muscle [19,29,30].

Thus, the main objective of this study was to examine the chemical composition, fatty acid profile, and amino acids of meat from Aubrac bulls and heifers raised in a semi-intensive system in Romania. Considering that the Aubrac cattle breed has gained increased interest among farmers, we believe it is necessary to highlight the qualitative properties of the beef obtained from these cattle to assist beef consumers. Although Aubrac cattle are internationally recognized for superior meat quality, research remains limited, particularly in Romania, where the breed has only recently been introduced and no detailed studies have yet been conducted. This highlights the need for an in-depth analysis to provide solid data on the nutritional profile of Aubrac beef, supporting informed choices by consumers and farmers. By delivering novel insights into the Aubrac breed, this study not only fills a gap in the literature but also contributes to sustainable cattle breeding strategies, reinforces the value of beef as a nutritional resource, and addresses important socio-economic demands.

## 2. Materials and Methods

### 2.1. Biological Material

**Figure 1:**
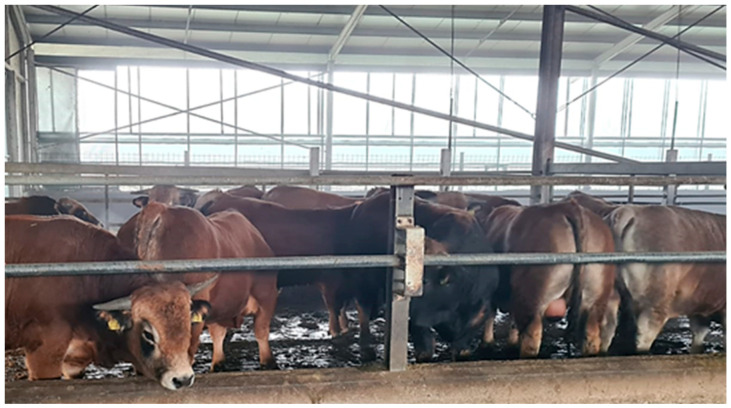
The Aubrac cattle breed on a farm in the southeast of Romania, in Calarasi county (original photography).

The study used Aubrac breed cattle, including both males and females, aged 15–18 months, raised in a semi-intensive system (Figure 1) on a farm in the southeast of Romania (Calarasi county). A total of 49 animals were included (27 males, 22 females) selected for fattening and subsequent slaughtering.

Animal diets were monitored in a controlled 300-hectare environment where all the forage was cultivated. The daily ratio, formulated to meet essential nutritional requirements and physiological limits for an average weight gain of 1200 g/day, consisted of roughage (natural hay, 18%), energy sources (sugar beet, 55%; corn, 16%), protein (soybean meal, 10%), and mineral supplements (calcium carbonate, dicalcium phosphate), maintained consistently throughout the three-month fattening period.

This ration provided 6.7 Beef Load Digestive Units (BLDUs), corresponding to a net energy (NE) requirement of 6.47 Mcal, including 542 g of digestible protein (PDI), 41.14 g of calcium (Ca), and 24.53 g of phosphorus (P), calculated according to INRA feeding standards. Fresh water was provided daily (30–50 L per animal). Total daily feed intake is represented in Table 1, amounting to 10.86 kg/animal/day.

Average daily gain (ADG) ranged from 839.0 ± 14.16 g/day to 985.2 ± 19.54, higher in males (Table 2). 

Average daily gain (ADG) is presented in grams, converted from kg/day by multiplying by 1000 [3,14].

Overall carcass yield was 57.7 ± 1.2% (Table 2), which indicates the ratio between carcass weight and live weight [4,13].

These results reflect feed utilization efficiency and productive performance under the applied semi-intensive management system.

Cattle were slaughtered at 18 months in the farm’s own slaughterhouse using a stunning method followed by exsanguination, in accordance with Regulation (EC) No. 1099/2009 and hygiene/food safety standards [31]. Slaughter procedures were supervised by trained personnel to ensure compliance with recognized animal welfare practices beyond legal requirements. Meat samples (~300–500 g) were collected from the *Longissimus dorsi* (13th rib, Sirloin), according to ISO 3100-1:1991, ensuring homogeneity and representativeness for chemical analyses [32]. Samples were properly prepared and stored for further analyses.

### 2.2. Assessment of Chemical Proximate Composition

Water content was assessed by the oven dehydration method, where the sample was dried at a temperature of +105 °C until a constant weight was achieved. The loss in weight during the process was used to calculate the moisture content, following the SR ISO 1442/1997 analytical protocol [33].

The determination of protein contents was performed using the Kjeldahl method, adapted for the Velp Scientifica system, comprising a DK6 digestion unit and a UDK7 distillation unit (VELP Scientifica Srl, Usmate, Italy; 981:10; AOAC Official Methods of Analysis/1990, which aligns with SR ISO 937/2008) [34].

Fat content determination was executed through the Soxhlet extraction method, which involves using an organic solvent to extract fats with the Velp Scientific-SER 148 apparatus (as per the manufacturer guidelines, AOAC Official Methods of Analysis/1990, compatible with SR ISO 1443/2008) [35].

The total mineral content was determined gravimetrically through the calcination method in an electric furnace, operating at a temperature of +550 °C, as outlined in the SR ISO 936:1998 standard [36].

### 2.3. Fatty Acid Profile Analysis and Lipid Profiles Implications

To determine the fatty acid profile in beef, an advanced method based on gas chromatography coupled with mass spectrometry (GC-MS) was employed, following the transformation of fatty acids into fatty acid methyl esters (FAME) [12,29]. First, lipids were extracted from the beef using a mixture of chloroform and methanol, according to a standard procedure that allowed for an efficient separation of lipids from the protein matrix. After evaporating the solvent, the extracted lipids underwent an esterification reaction using boron trifluoride (BF3) in methanol as a catalyst. The reaction took place at a temperature of 60 °C for 30 min, resulting in the formation of fatty acid methyl esters [29,34]. After the reaction, the methyl esters were separated using a gas chromatography system with a 30 m long and 0.25 mm internal diameter polar capillary column coated with polyethylene glycol (PEG). The temperature gradient was set to start at 100 °C and gradually increase to 250 °C, allowing for complete separation of the FAME. Helium was used as the carrier gas at a flow rate of 1.5 mL/min, and the split ratio was set at 40:1 [34,35]. The detection phase was conducted using mass spectrometry, where the methyl esters were fragmented and identified based on their molecular weight and characteristic fragments. The spectrometer operated with an ionization source at 105 °C and utilized target and qualifier ions for the precise identification of each compound. Quantification of the fatty acids was achieved by comparing retention times with a commercial reference standard (FAME Supelco 37 Mix) [34,36]. Thus, each fatty acid was expressed as a percentage of the total identified FAME, providing a detailed profile of the lipid composition in beef obtained from Aubrac cattle.

The fatty acid profile of Aubrac beef was analyzed to identify and quantify the lipophilic compounds present in this matrix. The following acids were determined: Capric Acid (C10:0), Lauric Acid (C12:0), Myristic Acid (C14:0), Myristoleic Acid (C14:1), Pentadecanoic Acid (C15:0), Palmitic Acid (C16:0), Palmitoleic Acid (C16:1), Margaric Acid (C17:0), Stearic Acid (C18:0), Oleic Acid (C18:1), Trans-Vaccenic Acid (C18:1), Linoleic Acid (C18:2), Alpha-Linolenic Acid (C18:3:cis:ω3), Arachidic Acid (C20:0), Eicosenoic Acid (C20:1), and C18:2 trans. Based on the results obtained, the following parameters were calculated: omega-3 fatty acids (Ω3 FAs), omega-6 fatty acids (Ω6 FAs), trans fatty acids (TFAs), saturated fatty acids (∑ SFAs = C10:0 + C12:0 + C14:0 + C15:0 + C16:0 + C17:0 + C18:0 +C20:0), monounsaturated fatty acids (∑ MUFAs = C14:1 + C16:1 + C18:1 + C20:1), polyunsaturated fatty acids (∑ PUFAs = C18:2 cis + C18:3 cis:ω3), and unsaturated fatty acids (∑ UFAs = MUFA + PUFA) [37]. Additionally, the ratios UFA/SFA and Ω6 FA/Ω3 FA were calculated, contributing to a better understanding of the lipid composition of beef, with significant implications for nutritional quality and health.

The atherogenic index (AI) and thrombogenic index (TI) of fats were determined using data from the FAME GC analysis of Aubrac beef, applying Equations (1) and (2) as described by Ulbricht and Southgate [38]:

AI = (C12:0 + C16:0 + 4 × C14:0)/[∑ MUFA + ∑ (n − 6) + ∑ (n − 3)]; (1)

TI = (C14:0 + C16:0 + C18:0)/[0.5 ∑ MUFA + 0.5 × ∑ (n − 6) + 3 × ∑ (n − 3) + ∑ (n − 3)/S(n − 6)].(2)

To calculate the ratio of hypocholesterolemic (h) to hypercholesterolemic (H) fatty acids, the Formula (3) proposed by Fernandez et al. [39] was applied:

h/H (hypocholesterolemic/Hypercholesterolemic) = (C18:1 + PUFA)/(C12:0 + C14:0 + C16:0).(3)

### 2.4. Amino Acid Profile Analysis and Protein Biological Value

Amino acid analysis of beef was conducted using a Mikrotechna AAA 881 automatic amino acid analyzer (Model 118/119 CL, CR). Prior to analysis, beef samples were hydrolyzed using a 6 M hydrochloric acid (HCl) solution and incubated at a temperature of 110 °C for 24 h [34]. This procedure converted cysteine into cysteic acid and methionine into methionine sulfone, thereby facilitating the accurate assessment of these sulphur-containing amino acids. In the case of tryptophan, it was determined after hydrolysis with sodium hydroxide (NaOH) at 110 °C for 22 h, according to the methodology described in the Official Methods of Analysis of the Association of Analytical Chemists [34,40]. Amino acid determination was expressed on a grams per 16 g nitrogen basis, equivalent to g/100 g of protein. This approach allows for a detailed evaluation of the amino acid profile, providing essential information for the nutritional analysis of beef [41].

Amino acids were grouped into the following categories for analysis: total amino acids (Total AAs), essential amino acids (eAAs), flavour-related amino acids (AAs), saccharinity-related amino acids (sAAs), and fragrant-related amino acids (frAAs) [42,43]. These groupings were used for statistical evaluation and calculation of principal component scores.

The qualitative differentiation of proteins was achieved through methods that enabled the classification of meat sample proteins and the identification of their complementary potential. These methods allowed for the assessment of protein value based on essential amino acid (AA) content, the proportion of exogenous amino acids (EAAs), the protein chemical score (CS), and the essential amino acid index (EAAI). After quantifying the essential amino acids in the samples, protein quality was evaluated by comparing its amino acid composition to that of a reference protein, according to the relation that renders the CS parameter (4):

(4) $$CS=\frac{AA\;content\;in\;the\;studied\;protein\;(meat\;sample\;protein)}{AA\;content\;in\;the\;standard\;protein\;(egg\;protein)}$$

Egg protein is commonly used as the standard or reference protein, as recognized by international scientific organizations such as the Food and Agriculture Organization of the United Nations (FAO), the World Health Organization (WHO), and the United Nations University (UNU). In this study, the nutritional value of proteins was calculated based on these established standards [37,38,39,40], which will be presented in the results section alongside our comparative evaluation.

The essential amino acid index (EAAI) was determined using Equation (5) and the chemical index values of the eight essential amino acids, following Oser’s methodology [44,45].

(5) $$\mathrm{EAAI}=\sqrt[{n}]{CS1\times CS2\times CS3\times \ldots \ldots .\times \;CSn}$$

Oser’s mathematical equation (relation 6) [45,46] was applied to determine the Biological Value (BV) of proteins, while the nutritional index (NI) of meat samples was calculated using Equation (7) proposed by Crisan and Sands (1978) [47].

BV = 1.09 (EAAI) − 11.7 (6)

(7) NI(%)=(EAAI×% protein)/100

### 2.5. Data Analysis

The obtained data were initially compiled in a Microsoft Excel database for efficient management and were subsequently subjected to detailed comparative analysis using SPSS (Statistical Package for the Social Sciences). For the interpretation and understanding of the results, SPSS was used to apply statistical methodologies such as ANOVA (Analysis of Variance) and general descriptive calculations. Additionally, graphs and supplementary analyses were generated, such as the calculation of eigenvalues and the creation of preference maps. These graphical analyses were performed using statistical procedures, such as Principal Component Analysis (PCA), with the SPSS v.20 software package (SPSS Inc., Chicago, IL, USA). A total of 27 replicates per sample were conducted for proximate chemical composition (water, proteins, lipids, and minerals), and 7 analytical replicates were performed for fatty acids and amino acids.

## 3. Results

### 3.1. Chemical Proximate Composition of Aubrac Beef

The results focus on comparing the chemical parameters of meat obtained from both males and females of Aubrac raised in a semi-intensive system, highlighting the differences and similarities between the two categories. Table 3 presents these results, providing valuable information about the composition of meat.

### 3.2. Fatty Acid Profiling of Aubrac Beef

The profile of fatty acids and the assessment of relevant indices are presented in Table 4. The analyses were conducted on the *Longissimus dorsi*, with samples obtained from both genders (males and females).

The fatty acids in beef are an important aspect of the meat’s chemical composition. We classify them into saturated and unsaturated fatty acids, and their proportion and types can influence the quality and nutritional characteristics of the meat. Observed sex-specific differences likely reflect hormonal regulation, with estrogen in females promoting higher monounsaturated fatty acid accumulation and testosterone in males favoring saturated fatty acid deposition, which provides a mechanistic explanation for variations in meat quality.

**Figure 2:**
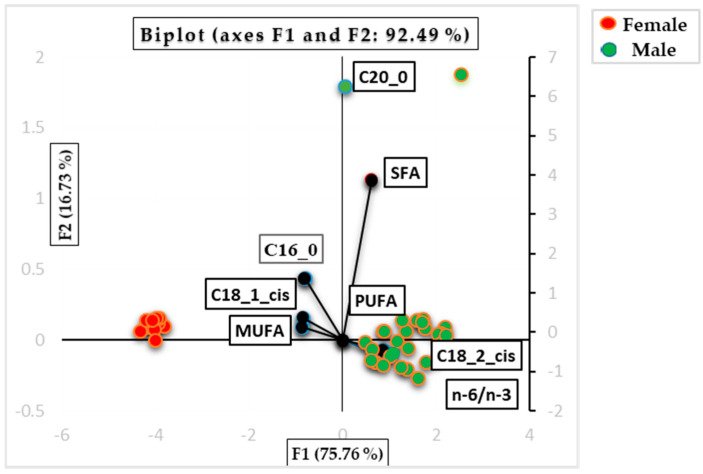
PCA factor-score biplots explaining the two component scores (FI and F2) for the sample of Aubrac beef and display similarities between samples according to their fatty acids concentration (SFA = saturated fatty acid; MUFA = monounsaturated fatty acid; PUFA = polyunsaturated fatty acid; n−6/n−3 = ratio omega 6/omega 3).

Figure 2 illustrates the PCA factor score biplots, which explain the two main components (F1 and F2) derived from the analysis. These biplots reveal the similarities and groupings among samples based on their fatty acid concentrations, including saturated fatty acids (SFAs), monounsaturated fatty acids (MUFAs), polyunsaturated fatty acids (PUFAs), and the omega-3 to omega-6 ratio (n−3/n−6). The data are further categorized by gender, with red bullets representing female samples and green bullets representing male samples.

### 3.3. Amino Acid Profiling of Aubrac Beef

Table 5 displays the amino acid profile and the evaluation of relevant indices. Samples of *Longissimus dorsi* from both genders were used for the analyses.

To assess the nutritional value of the investigated meat protein samples, the amino acid content was expressed as grams of amino acids per 100 g of protein. This approach allows for direct comparison with FAO/WHO standards for different consumer categories and facilitates the nutritional evaluation of meat proteins (Table 6).

**Figure 3:**
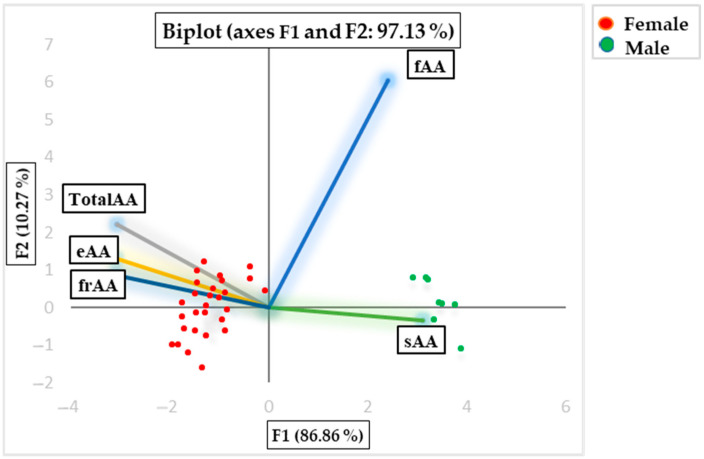
PCA factor-score biplots, explaining the two component scores (FI and F2) for the sample of Aubrac beef and display similarities between samples according to their amino acids concentration (Total AA = total amino acid; eAA = essential amino acid; fAA = flavor-related amino acid; sAA = sacharinity-related AA; frAA = fragrant-related amino acid).

Figure 3 presents the PCA factor score biplots, which explain the two principal component scores (F1 and F2) for the Aubrac beef samples. These plots display the similarities between samples based on their amino acid concentrations. The analysis focuses on various categories of amino acids, including total amino acids (Total AAs), essential amino acids (eAAs), flavor-related amino acids (fAAs), saccharinity-related amino acids (sAAs), and fragrance-related amino acids (frAAs). The samples are further categorized by gender, with red bullets representing female samples and green bullets representing male samples. The biplots provide insight into how different amino acid groups contribute to the variability in the beef samples.

## 4. Discussion

### 4.1. Chemical Proximate Composition of Aubrac Beef

The evaluation of the chemical composition of the *Longissimus dorsi* muscle from Aubrac cattle revealed significant results for water, dry matter, proteins, fats, and minerals (Table 3). The water content was 75% in males and 71.84% in females, suggesting greater juiciness in males, a trait valued by consumers. These results are consistent with previous studies [48,49] and are supported by Junka et al. (2017), who reported 73.50% water in Aubrac beef [50]. Compared with Angus (73–74%) and Charolais (72–73%), Aubrac beef exhibits slightly higher moisture, particularly in males, potentially enhancing juiciness [9,30]. The percentage of dry matter was 25% for males and 28.16% for females, indicating a higher density of muscle tissue in females, which may influence meat texture and quality. These dates are consistent with the studies by Bakharev et al. (2020), who reported a similar dry matter content of 25.42% for beef [28,51].

The protein content was 21.85% in males and 21.38% in females, confirming the breed’s high nutritional value. Chen et al. (2022) reported 20–24% protein in beef [52]. Comparable data for Angus (20–22%) and Charolais (21–23%) indicate that Aubrac protein content aligns with these major breeds, supporting its high nutritional quality [9,30]. Fat content was significantly higher in females (4.80%) than in males (1.88), consistent with industry trends where females generally exhibit higher fat content, enhancing juiciness and flavor. Blanco et al. (2020) reported 4.91% fat in beef [33,53]. Besides enhancing juiciness and flavor, fats also contribute to the aromatic profile appreciated by consumers [40,54]. The mineral content was 1.05% in males and 0.82% in females. These results are in line with previous studies highlighting beef as an important source of essential minerals, such as zinc and iron, with comparable values also reported for Aubrac [51].

The analysis of the chemical composition of the *Longissimus dorsi* from Aubrac cattle reveals distinct differences between genders in water, dry matter, protein, fat, and mineral content. Males show higher water and mineral contents, contributing to juiciness and nutritional value, while females present greater dry matter and fat, enhancing flavor and tenderness. These findings emphasize Aubrac’s balanced nutritional profile and its relevance for meat production. These sex-related differences suggest that rearing and feeding strategies could be adjusted to optimize meat quality, with males prioritized for juiciness and nutritional value, and females for flavor and tenderness.

### 4.2. Fatty Acid Profiling of Aubrac Beef

Fatty acids are key determinants of meat quality and nutritional value, classified as saturated (SFA) or unsaturated (MUFA, PUFA) [55,56,57].

According to Table 4, oleic acid was the most abundant, with values of 34.02 g/100 g in males and 39.31 g/100 g in females, showing significant differences between genders. This predominance of oleic acid indicates a favorable lipid profile, consistent with health-promoting effects of MUFA. The observed sex-related differences may be explained by biological factors such as metabolism and sex hormones. We hypothesize that sex-specific hormones, particularly testosterone in males and estrogen in females, play a key role in regulating adipose tissue accumulation and distribution in Aubrac beef.

Saturated fatty acids (SFAs) showed significant sex-related differences: males had 49.75%, while females had 46.85%. Conversely, monounsaturated fatty acids (MUFAs) were lower in males (37.21%) and higher in females (44.45%). Polyunsaturated fatty acids (PUFAs) also differed, with males at 4.42% and females at 2.66%. Overall, unsaturated fatty acids were higher in females (47.11%) than in males (41.64%).

Additionally, total fatty acids in the analyzed meat samples were dominated by saturated fatty acids (SFAs, 48.95%), followed by monounsaturated fatty acids (MUFAs, 39.11%) and polyunsaturated fatty acids (PUFAs, 3.96%). This profile is typical for beef and aligns with previous reports. For example, Atillo L. Mordenti (2019) reported 49.86% SFA, 44.83% MUFA, and 3.80% PUFA in *Longissimus dorsi* from Charolais x Aubrac [58]. Similarly, D. Bures et al. (2006) reported SFA 48.87–53.30%, MUFA 39.29–39.56%, and PUFA 7.23–8.31% [59,60]. Aubrac beef exhibited a slightly lower percentage of saturated fatty acids (49.86%) compared to other breeds (Charolais—53.3%) [61,62,63].

The beef from the Aubrac breed exhibits a balanced lipid profile, as evidenced by essential health-related fatty acid indices (Table 4). The Atherogenic Index (AI), with an overall value of 0.821, suggests a moderate potential for promoting atherosclerotic plaque formation. Calculated at 27.11% the proportion (overall) of hypercholesterolemic fatty acids (HFAs) indicates a moderate presence of saturated fatty acids (C12:0, C14:0, C16:0). This is counterbalanced by hypocholesterolemic fatty acids (hFAs) (5.89%, including C:18:1 and polyunsaturated fatty acids). The hypocholesterolemic/hypercholesterolemic ratio (h/H), with a value of 0.217, indicates moderate lipid quality. Consequently, Aubrac beef can be considered a nutritionally valuable option, suitable for general consumption. It is a competitive choice among red meats, suitable for inclusion in a balanced diet.

Figure 2 illustrates the score biplots for the first two principal components (F1 and F2), which together accounted for 92.49% of the total variation. F1 accounted for 75.76% of the total variance and was primarily positively associated with C18:2:cis (0.981), C20:0 (0.065), n−6/n−3 (0.978), SFA (0.694), and PUFA (0.974), and negatively associated with C16:0 (−0.922), C18:1cis (−0.955), and MUFA (−0.973). F2 explained 16.73% of the total variance and was positively correlated with C16:0 (0.233), C18:1:cis (0.084), C20:0 (0.952), SFA (0.601), and MUFA (0.050) and negatively correlated with C18:2:cis (−0.042), n−6/n−3 (−0.060), and PUFA (−0.037). The observed sex-related differences in fatty acid composition may be influenced by sex-specific hormonal regulation, with testosterone in males and estrogen in females affecting lipid metabolism and fatty acid deposition. Differential activity of key enzymes involved in fatty acid synthesis and desaturation, such as stearoyl-CoA desaturase and fatty acid synthase, may also contribute to these distinct lipid profiles. Together, these mechanisms suggest that hormonal signaling and metabolic enzyme activity underlie the sex-dependent variations in fatty acid composition, highlighting their nutritional and physiological relevance [64,65,66,67].

Understanding these differences may help producers tailor diets or management practices to enhance beneficial fatty acids in each gender, supporting both nutritional quality and consumer preferences. The observed sex-specific amino acid profiles may inform herd management and product differentiation, with males potentially prioritized for protein-rich products and females for cuts emphasizing flavor and tenderness.

### 4.3. Amino Acid Profiling of Aubrac Beef

Amino acids are the fundamental components of proteins and can vary depending on species, breed, and diet [68,69]. Assessment of the amino acid profile provides precise information on meat quality and nutritional value [70,71,72].

As shown in Table 5, there are highly significant differences between genders in *Longissimus dorsi* of Aubrac for aspartic acid, glutamic acid, alanine, arginine, phenylalanine, glycine, isoleucine, histidine, leucine, lysine, methionine, proline, serine, tyrosine, threonine, and valine. Additionally, significant differences between genders were observed for cysteine (0.29% M, 0.27% F) and tryptophan (3.06% M, 2.85% F), whereas variations in hydroxyproline were insignificant (0.01% M; 0.01% F). The values obtained in this study are consistent with those reported for other meat-specialized breeds. Alekseeva (2006) reported values of 2.03 g/100 g for aspartic acid, 0.22 g/100 g for cysteine, and 0.91 g/100 g for tyrosine in Angus beef, while the present study on Aubrac beef found 2.56 g/100 g (aspartic acid), 0.28 g/100 g (cysteine), and 1.00 g/100 g (tyrosine) [73]. The differences observed between breeds are relatively small, suggesting that both Aubrac and Angus exhibit similar amino acid profiles for aspartic acid, cysteine, and tyrosine. Minor variations may arise from genetics, nutrition, and management practices, influencing amino acid composition despite similar production purposes [73,74].

Regarding total amino acid content, highly significant differences exist between genders, with males averaging 30.59% and females 25.97%. Animals were raised under identical semi-intensive conditions and fed the same diet, eliminating environmental and nutritional variability [75]. These differences likely reflect intrinsic physiological gender-related variations. Males may have a natural predisposition for higher protein content, independent of growth conditions and diet [76,77].

The results in Table 5 show significant differences between males and females regarding the content of essential and non-essential amino acids in the *Longissimus dorsi* muscle. Males have a higher content, with 15.05% essential amino acids and 15.56% non-essential amino acids, compared to females, who have 12.68% essential amino acids and 13.28% non-essential amino acids. These differences suggest a richer protein composition in males, which could be related to physiological factors such as more intense muscle growth [76].

Protein or amino acid deficiency can cause health problems in children, adults, and the elderly [77]. Balanced dietary protein intake is essential to prevent deficiencies or excesses [78].

The biological value of the protein in the *Longissimus dorsi* muscle from Aubrac was estimated by analyzing the essential and semi-essential amino acid content (g/100 g protein). Results were compared with FAO and WHO reference values for three age-identified consumer categories (children, youth, and adults), according to the data presented in Table 2. These comparisons revealed higher values for the eight essential amino acids (valine, isoleucine, leucine, lysine, methionine, threonine, tryptophan, phenylalanine) and the two semi-essential amino acids (cystine and tyrosine) in bovine *Longissimus dorsi* muscle for all three consumer groups. The relatively high tryptophan values observed in Aubrac beef compared to literature reports may be attributed to the breed’s genetic characteristics and the specific diet and nutritional management of the animals. Additionally, the slightly higher values observed in males could be influenced by sex-specific hormonal effects on protein metabolism. These factors together likely explain the elevated tryptophan content observed in our study.

According to the specialized literature, the calculation of the Oser index or the EAA index [44,77] demonstrates that *Longissimus dorsi* muscle contains high-quality proteins, as the proportions of essential and semi-essential amino acids exceed 100% of the standard protein, being 160.25% for children, 197.43% for youth, and 321.49% for adults (Table 6). This was further supported by the biological value (BV) and the nutritional index (NI). The notably high EAAI and BV (e.g., 321.49% and 338.73% for adults) reflect the exceptionally high content of essential amino acids in Aubrac beef. These values are biologically plausible, given the breed’s genetic potential and semi-intensive feeding system, indicating superior protein quality compared with standard reference proteins.

Previous studies on the nutritional quality of the *Longissimus dorsi* muscle have shown that protein and amino acid content can vary depending on species, breed, and management. Genetic factors largely determine meat composition, while nutritional and management practices can further influence muscle fiber chemical structure [79,80,81,82].

Amino acids from beef obtained from females and males were analyzed, with statistical results serving as the foundation for applying Principal Component Analysis. Figure 3 illustrates the score biplots for the first two principal components (F1 and F2), which together accounted for 97.13% of the total variation. F1 accounted for 86.86% of the total variance and was primarily positively associated with fAA (0.757), sAA (0.979), and negatively associated with total AA (−0.963), eAA (−0.975), and frAA (−0.966). F2 explained 10.27% of the total variance and was positively correlated with total AA (0.240), eAA (0.142), fAA (0.653), and frAA (0.093) and negatively correlated with sAA (−0.036).

## 5. Conclusions

The findings of this research reveal significant gender-related differences in beef chemical composition, including in the ratio of unsaturated/saturated fatty acids and the Ω6 FA/Ω3 FA ratio. Total amino acid content also showed higher average values in males. Nutritional evaluation demonstrated that the Longissimus dorsi muscle contains high-quality proteins, with essential and semi-essential amino acids present in proportions exceeding standard reference proteins. These results indicate the superior protein quality of Aubrac beef, reflecting the breed’s genetic potential and semi-intensive feeding system. The findings can guide farmers in selection and breeding decisions, contributing to the production of high-quality animals and efficient, profitable outputs. They can also support the adaptation of production to market demands and the development of targeted marketing strategies. Overall, this study advances scientific knowledge and provides a foundation for future research on nutrition, genetics, and processing technologies to further improve the quality and value of Aubrac beef.

## Figures and Tables

**Table 1:** Daily nutritional requirements and provided diets for growing beef cattle in a semi-intensive system (15–18 months).

Daily Ration	Kg Feed	Nutritional Requirements				
		BLDU *	NE **	PDI *** (g)	Ca (g)	P (g)
		6.7	6.46	542	42	25
Daily average yield		1200 g				
Covered						
Natural hay	2.0	2.22	1.26	106	10.2	5.2
Sugar beet	6.0	0.84	1.95	48	4.74	3.6
Soybean meal	1.1	-	1.41	324.5	3.63	7.54
Corn	1.7	-	1.85	107.1	1.17	6.34
Calcium carbonate	0.05	-	-	-	19.0	-
Dicalcium phosphate	0.01	-	-	-	2.4	1.85
Daily covered	10.86	3.06	6.47	585.6	41.14	24.53

* BLDU = Beef load digestive units (maximum physiological threshold); ** NE = Net energy requirement of growing beef cattle; *** PDI = digestible protein at the intestinal level.

**Table 2:** Productive Qualities and Carcass Characteristics of Aubrac cattle at 18 months.

Productive Qualities	Male (n = 27)	Female (n = 22)	Overall
Parameters	$\bar{X}$ ± SD	$\bar{X}$ ± SD	$\bar{X}$ ± SD
Weight at 18 months (kg)	627.27 ± 29.45	564.76 ± 20.77	610.82 ± 38.94
Average daily gain (g/day)	985.2 ± 19.54	839.0 ± 14.16	912 ± 17.04
Carcass weight (kg)	376.36 ± 19.62	313.72 ± 12.84	354.04 ± 16.57
Carcass yield (%)	60 ± 1.36	55.4 ± 1.02	57.7 ± 1.2

**Table 3:** Chemical proximate composition (%) of *Longissimus dorsi* muscle.

Gender		
%	Male	Female
	$\bar{X}$ ± SD	$\bar{X}$ ± SD
Water	75.00 ± 0.43 ^a^	71.84 ± 0.19 ^b^
Dry Matter	25.00 ± 0.43 ^b^	28.16 ± 0.19 ^a^
Protein	21.85 ± 0.20 ^a^	21.38 ± 0.41 ^b^
Total Fat	1.88 ± 0.06 ^b^	4.80 ± 0.10 ^a^
Minerals	1.05 ± 0.06 ^a^	0.82 ± 0.06 ^b^

Different superscript letters (a,b) within the same row indicate significant differences between genders (*p* < 0.05).

**Table 4:** Fatty acid profile (% of total lipids) of Aubrac cattle beef (*Longisimus dorsi* muscle).

Fatty Acid (%)	Gender			
	Male (n = 27)	Female (n = 22)	Significance	Overall
	X ± SD	X ± SD		X ± SD
Capric—C10:0	0.51 ± 0.09	0.10 ± 0.02	***	0.40 ± 0.20
Lauric—C12:0	0.50 ± 0.11	0.12 ± 0.02	***	0.40 ± 0.19
Myristic—C14:0	3.26 ± 0.07	3.36 ± 0.10	**	3.28 ± 0.09
Myristoleic—C14:1	0.62 ± 0.11	0.64 ± 0.05	ns	0.63 ± 0.09
Pentadecanoic—C15:0	0.47 ± 0.05	0.50 ± 0.06	ns	0.48 ± 0.05
Palmitic—C16:0	21.72 ± 0.98	28.23 ± 0.74	***	23.43 ± 3.05
Palmitoleic—C16:1	2.39 ± 0.36	4.38 ± 0.34	***	2.92 ± 0.95
Margaric—C17:0	1.04 ± 0.07	0.98 ± 0.05	*	1.02 ± 0.07
Stearic—C18:0	22.15 ± 1.24	13.45 ± 0.61	***	9.86 ± 4.04
Oleic—C18:1:cis	34.02 ± 1.10	39.31 ± 0.54	***	35.41 ± 2.56
Trans-vaccenic—C18:1	1.95 ± 0.17	1.86 ± 0.09	ns	1.93 ± 0.16
Linoleic—C18:2:cis	3.99 ± 0.28	2.10 ± 0.04	***	3.50 ± 0.88
Alpha-linolenic C18:3:cis:ω3	0.44 ± 0.03	0.56 ± 0.09	***	0.47 ± 0.07
Arachidic—C20:0	0.08 ± 0.14	0.09 ± 0.01	ns	0.09 ± 0.12
Eicosenoic—C20:1	0.17 ± 0.01	0.12 ± 0.01	***	0.16 ± 0.02
C18:2:trans	2.52 ± 0.28	0.14 ± 0.01	***	1.89 ± 1.09
Ω3 FA	0.44 ± 0.03	0.56 ± 0.09	***	0.47 ± 0.07
Ω6 FA	6.51 ± 0.45	2.23 ± 0.05	***	5.39 ± 1.95
∑ TFA	2.67 ± 0.16	2.06 ± 0.18	***	2.51 ± 0.32
∑ SFA	49.73 ± 1.90	46.82 ± 0.38	***	48.96 ± 2.08
∑ MUFA	37.21 ± 1.15	44.45 ± 0.61	***	39.11 ± 3.39
∑ PUFA	4.43 ± 0.29	2.66 ± 0.10	***	3.96 ± 0.83
∑ UFA	41.64 ± 1.15	47.11 ± 0.65	***	43.08 ± 2.65
^1^ UFA/SFA	0.84 ± 0.04	1.01 ± 0.02	***	0.88 ± 0.08
^2^ Ω6 FA/Ω3 FA	14.93 ± 1.24	4.06 ± 0.65	***	12.07 ± 4.97
AI	0.798	0.885	***	0.821 ± 0.05
TI	2.03	1.74	***	1.54 ± 0.17
HFA	25.48	31.71	***	27.11 ± 3.15
hFa	6.38	4.52	***	5.89 ± 0.93
h/H	0.250	0.142	***	0.217 ± 0.06

Significance: *** *p* ≤ 0.001; ** *p* ≤ 0.01; * *p* ≤ 0.05; ns—not significant; n = number of samples. Ω3 FA—omega-3 fatty acid; Ω6 FA—omega-6 fatty acid; TFA—trans fatty acid; SFA—saturated fatty acid; MUFA—monounsaturated fatty acid; PUFA—polyunsaturated fatty acid; ^1^ UFA/SFA—ratio of unsaturated to saturated fatty acids; ^2^ Ω6 FA/Ω3 FA—ratio of omega-6 to omega-3 fatty acids; UFA—unsaturated fatty acid. AI—atherogenic index; TI—thrombogenic index; HFA—hypercholesterolemic fatty acid (C12:0 + C14:0 + C16:0); hFa—hypocholesterolemic fatty acid (C18:1 + polyunsaturated FA); h/H—ratio of hFa to HFA.

**Table 5:** Amino acid profile (g/100 g) of Aubrac cattle beef (*Longisimus dorsi* muscle).

Amino Acids	Gender			
	Male (n = 27)	Female (n = 22)	Significance	Overall
	X ± SD	X ± SD		X ± SD
Aspartic acid	2.67 ± 0.18	2.24 ± 0.11	***	2.56 ± 0.25
Glutamic acid	4.31 ± 0.19	3.72 ± 0.20	***	4.16 ± 0.33
Alanine	1.61 ± 0.10	1.42 ± 0.13	***	1.56 ± 0.14
Arginine	2.12 ± 0.12	1.86 ± 0.08	***	2.05 ± 0.16
Cystine + Cysteine	0.29 ± 0.02	0.27 ± 0.02	*	0.28 ± 0.02
Phenylalanine	1.12 ± 0.09	0.93 ± 0.09	***	1.07 ± 0.12
Glycine	1.19 ± 0.11	1.00 ± 0.07	***	1.14 ± 0.13
Hydroxyproline	0.01 ± 0.00	0.01 ± 0.00	ns	0.01 ± 0.00
Isoleucine	1.33 ± 0.11	1.12 ± 0.12	***	1.28 ± 0.14
Histidine	1.17 ± 0.10	0.95 ± 0.09	***	1.11 ± 0.13
Leucine	2.35 ± 0.14	1.89 ± 0.11	***	2.23 ± 0.25
Lysine	2.60 ± 0.16	2.15 ± 0.15	***	2.48 ± 0.26
Methionine	0.75 ± 0.06	0.60 ± 0.06	***	0.71 ± 0.08
Proline	1.10 ± 0.10	0.87 ± 0.07	***	1.04 ± 0.14
Serine	1.21 ± 0.11	1.02 ± 0.07	***	1.16 ± 0.13
Tyrosine	1.04 ± 0.10	0.88 ± 0.06	***	1.00 ± 0.12
Threonine	1.30 ± 0.08	1.09 ± 0.11	***	1.25 ± 0.13
Tryptophan (total)	3.06 ± 0.25	2.85 ± 0.27	*	3.00 ± 0.27
Valine	1.38 ± 0.10	1.11 ± 0.09	***	1.31 ± 0.16
**^1^ Total amino acids**	30.59 ± 0.49	25.97 ± 0.64	***	29.37 ± 2.13
**^2^** **EAA%**	15.05%	12.68%		
**^3^** **NEAA%**	15.56%	13.28%		

Significance: *** *p* ≤ 0.001; * 0.01 < *p* ≤ 0.05; ns *p* > 0.05; n = number of samples. ^1^ = total amino acids; ^2^ = essential amino acids (EAA%); ^3^ = non-essential amino acids (NEAA%).

**Table 6:** The amino acid content (g/100 g protein) of the meat sample taken from the *Longissimus dorsi* and the reference proteins.

Amino Acids	AA Content (g/100 g Protein)	FAO/WHO Reference Protein		
		Standard 1 Children	Standard 2 Youth	Standard 3 Adults
Isoleucine	5.92	4.6	4.0	3.0
Leucine	10.31	9.3	7.0	4.4
Lysine	11.47	6.6	5.5	3.1
Methionine + Cystine	4.58	4.2	3.5	2.7
Phenylalanine + Tyrosine	9.57	7.2	6.0	3.3
Threonine	5.78	4.3	4.0	2.6
Tryptophan	13.88	1.7	1.0	0.6
Valine	6.06	5.5	5.0	2.3
EAA (g/16 g N)	67.58	43.4	36.0	22.0
Amino acids	Nutritional evaluation of the proteins	Standard 1	Standard 2	Standard 3
Isoleucine		128.71	148.01	197.35
Leucine		110.91	147.35	234.42
Lysine		173.80	208.56	370.03
Methionine + Cystine		109.03	130.83	169.60
Phenylalanine + Tyrosine		132.98	159.57	290.14
Threonine		134.46	144.54	222.37
Tryptophan		816.24	1387.60	2312.67
Valine		110.17	121.18	263.44
EAAI (%)		160.25	197.43	321.49
BV		162.97	203.50	338.73
NI (%)		34.65	42.68	69.51

AA = amino acid; EAA = exogenous amino acid. EAAI (%) = essential amino acid index; BV = biological value; NI (%) = nutritional index.

## Data Availability

The data in this study are available upon request from the first author and the corresponding authors.
